# Inhibition of histone deacetylase (HDAC) by 4-phenylbutyrate results in increased junctional conductance between rat corpora smooth muscle cells

**DOI:** 10.3389/fphar.2015.00009

**Published:** 2015-02-03

**Authors:** Hong Zhan Wang, Barbara Rosati, Chris Gordon, Virginijus Valiunas, David McKinnon, Ira S. Cohen, Peter R. Brink

**Affiliations:** ^1^Department of Physiology and Biophysics, Stony Brook UniversityStony Brook, NY, USA; ^2^Department of Physiology and Biophysics, Molecular Cardiology Institute, Stony Brook UniversityStony Brook, NY, USA; ^3^Department of Neurobiology and Behavior, Stony Brook UniversityStony Brook, NY, USA

**Keywords:** 4-phenylbutyrate, connexin43, cell to cell coupling, patch clamp, protein expression

## Abstract

4-phenylbutyrate (4-PB) has been shown to increase the protein content in a number of cells types. One such protein is Connexin43 (Cx43). We show here that 4-phenylbutyrate exposure results in significantly elevated cell to cell coupling, as determined by dual whole cell patch clamp. Incubation with 5 mM 4PB for 24 h or more nearly doubles junctional conductance. Interestingly, mRNA levels for Cx43 declined with exposure to 4-PB while western blot analysis revealed not significant change in protein levels. These data are most consistent with stabilization of the existing Cx43 pool or alterations in the number of functional channels within an existing pool of active and silent channels. These data represent a baseline for testing the efficacy of increased connexin mediated coupling in a variety of multicellular functions including erectile function.

## Introduction

Corpora Cavernosa smooth muscle cells form a functional syncytium as a consequence of connexin derived gap junction channels connecting cell interiors (Harris, 2001). The most ubiquitously expressed connexin gene in corpora smooth muscle is Connexin43 (Cx43) (Asklund et al., 2004). Reduced expression of connexins has been implicated as a major contributor to impaired function with reduced expression of connexins (Melman and Christ, 2001). Further, a number of studies strongly suggest that Cx43 expression and gap junction mediated coupling are essential for relaxation of corpora smooth muscle cells and hence erectile response (Christ and Lue, 2004; Pointis, 2006; Fu et al., 2008; Suadicani et al., 2009).

A relevant question becomes: can up-regulation or enhanced functionality of Cx43 lessen erectile dysfunction? To assess this possibility it is first necessary to assess Cx43 expression and junctional conductance in corpora smooth muscle cell pairs.

Up-regulation of connexin expression can be accomplished with exposure to mild hyperthermia (VanSlyke and Musil, 2005). The expression of adhesion molecules such as alpha and beta catenin and cadherin can also affect connexin expression (Jongen et al., 1991; Prowse et al., 1997; Wei et al., 2005) and organ function (Ferreira-Cornwell et al., 2002; Li et al., 2006). The antiarrhythmic peptide ZP123 (Rotigaptide) has also been to shown to increase junctional conductance in cardiac myocyte pairs as much as 69% within minutes (Eloff et al., 2003; Xing et al., 2003; Axelsen et al., 2006) but it has not been shown to affect expression levels suggesting that it acts to recruit channels from a silent pool. The idea of a silent reservoir is based on the fact that active Cx43 channels in corpora have been shown to have open probabilities approaching between 0.5 and unity (Brink et al., 1996; Christ and Brink, 1999).

Another choice is chemical stimulation with 4-PB (Asklund et al., 2004) that inhibits histone deacetylases (HDACs), enzymes associated with inactivation of gene transcription (Perlmutter, 2002; Asklund et al., 2004; Iwasaki et al., 2006). 4-PB is an FDA approved drug that can be administered orally and is currently being tested for effectiveness in spinal muscular atrophy (Wirth et al., 2006), cystic fibrosis (Singh et al., 2006), and cancer (Tang et al., 2004). *In vitro* studies on cancer cell lines are of particular interest as they suggest Cx43 up-regulation results in channel recruitment. Asklund et al. (2004) found that 5 mM 4-PB increased Cx43 expression two- to five-fold with exposure times of hours to days in human glioblastoma cells and also reduced proliferation rate two- to three-fold and Khan et al. (2007) found that 4-PB induced apoptosis in human glioma cells. 4-PB has also been shown to elevate Cx43 expression in both Cx43 transfected cells (HeLa) and cells that endogenously express Cx43 (HEK-293) (Kaufman et al., 2013). All three studies also noted that exposure to 4-PB enhanced fluorescent dye transfer between cells consistent with an increase in the number of functional gap junction channels linking cell interiors.

We have used dual whole cell patch clamp to monitor junctional conductance in rat corpora cavernosa cell pairs to determine if 4PB exposure caused junctional conductance to increase between corpora smooth muscle cell pairs. We also monitored 4-PB effects on Cx43 expression using real-time PCR and Western Blot. We observed no significant change Cx43 protein levels contrary to previous reports (Asklund et al., 2004; Khan et al., 2007; Kaufman et al., 2013) but found that junctional conductance increased 1.4-fold after 24 h of exposure and attained a maximum increase of 1.8-fold after 72 h of exposure to 4PB. Voltage dependence was not affected by 4PB.

## Methods

### Cell source

Rat corporal tissue was dissected from the rat penis as previously described (Rehman et al., 1997; Wang et al., 2001). Explant cultures of rat corporeal cells were prepared as follows. Short-term cultures, i.e., passages 0–2, were prepared from freshly isolated rat corporeal myocytes (Wang et al., 2001) Cells were plated on 35 mm dishes and allowed to attach and proliferate for 1–2 days (passage 0) or they were split upon confluence (passage 1) prior to trypsinization and resuspension onto cover dishes for patch clamp analysis (Rehman et al., 1997; Wang et al., 2001).

### Patch clamp

Dual whole cell patch clamp of cell pairs was used as previously described (Wang et al., 2001; Valiunas et al., 2002). Media was exchanged for a buffered solution containing 150 mM NaCl, 4 mM KCl, 1 mM MgCl_2_, 5 mM HEPES, 2 mM pyruvate, and 2 mM CaCl_2_, pH 7.2 containing no 4-PB. The pipette solution contained 130 mM KCl, 10 mM NaCl, 1 mM MgCl_2_, 10 mM egtazic acid and 1 mM CaCl_2_, pH 7.0. Pipette resistance varied from 2 to 5 MΩ. Step protocols to monitor junctional conductance were as previously described (Wang et al., 2001). Data analysis was performed as previously described to determine voltage dependence, and junctional conductance (Valiunas et al., 2001; Wang et al., 2010).

### Western blot

Rat corpora smooth muscle cells or rat mesenchymal stem cells were exposed to 4-PB at concentrations of 0, 1, or 5 mM for 48 h. Cells were collected from cultures by first washing then scraping in cold PBS. Cell suspensions were centrifuged at 14,000 rpm at 4°C for 10 min then the supernatants were removed. The pellets were resuspended in cold RIPA (Radio-Immnuoprecipitation Assay) buffer (R0278, Sigma), protease inhibitor cocktail (AEBSF, Aprotinin, Bestatin hydrochloride, E-64, EDTA, Leupeptin) (P2714, Sigma), sodium orthovanadate (S-6508, Sigma) and PMSF (P-7626, Sigma). Samples were kept on wet ice during the lysis step for ~30 min and frequently vortexed. Samples were then centrifuged in an Eppendorf microfuge at 4°C, 14,000 rpm for 10 min, supernatants were transferred to new pre-chilled microtubes. Protein concentration of the samples was determined by the Bradford assay. Volumes containing 30 μg of total protein for each dose point were mixed with equal volumes of Laemmli sample buffer (161-0737, Bio Rad) containing β-mercaptoethanol and boiled for 5 min at 95°C. All samples were cooled then centrifuged briefly before being loaded on a SDS-polacrylamide gel (4% stacking gel, 10% separating gel). After separation by electrophoresis at 115 V for 90 min in tris-glycine/SDS buffer, proteins were transferred to Immobilon-P membrane (Millipore) by electrophoresis at 100 V for 60 min in tris-glycine/methanol buffer. Non-specific antibody binding was blocked for 1 h at RT in 5% Blotting Grade Blocker non-fat dry milk (Bio Rad) dissolved in 1x TBST. The membrane was washed briefly in 1x TBST (Wang et al., 2010).

*For Cx43*: a 43 kDa protein was probed for by incubating the membrane with the anti-connexin 43 antibody (C 6219, Sigma) at 1:8000 in 1% milk for 1 h at room temperature. After washing the membrane well with 1x TBST the membrane was incubated for 1 h at room temperature with goat anti-rabbit IgG-HRP (sc-2004, Santa Cruz) at 1:10,000 in 1% milk. After washing the membrane well with 1x TBST the secondary antibody was detected using SuperSignal West Femto Maximum Sensitivity Substrate (34095, Pierce) and images obtained by exposing the membrane to HyBlot CL Autoradiography Film (E3012, Denville Scientific).

For Tubulin a 55 kDa protein was probed for by incubating the membrane for 1 h at room temperature with Anti-α-Tubulin (sc-8035, Santa Cruz) at 1:1000 in 1% milk. After washing the membrane well with 1x TBST the membrane was incubated for 1 h at room temperature with goat anti-mouse IgG-HRP (1858413, Pierce) at 1:10,000 in 1% milk. After washing the membrane well with 1x TBST the secondary antibody was detected using SuperSignal West Femto Maximum Sensitivity Substrate (34095, Pierce) and images obtained by exposing the membrane to HyBlot CL Autoradiography Film.

The Western blot results were evaluated by making comparisons between bands in different lanes. Quantification of the intensity of the protein bands seen on the x-ray film was done using the free NIH ImageJ software. First a digital image of the film was made with a scanner then converted to gray scale. A rectangular box was drawn around the first band and selected, the box was then moved to each of the other bands and again selected. Once all were selected the bands were then plotted which generated a graphical depiction of band intensities. Using the draw line tool each band histogram was marked off at its base. The magic wand tool was clicked inside each histogram, after each peak was selected, label peaks was clicked which generated numerical values of band intensities. The same procedure was done for the loading control and the experimental data was normalized resulting in adjusted density values.

### RNA extraction and real-time PCR

Total RNA was extracted from control and 4-PB treated corpora smooth muscle cell cultures (RNeasy Miniprep Kit, Qiagen, Valencia, CA). Three independent 100-mm diameter culture dishes were used for each experimental condition. The RNA samples were quantitated by optical density measurement and then diluted to the same nominal concentration. A second round of optical density measurement was performed to confirm the accuracy of this step. Five milligrams of RNA was used for cDNA synthesis (Superscript III reverse transcriptase, Life Technologies). The cDNA samples were diluted 1:16 prior to real-time PCR analysis.

Real-time PCR was performed with a StepOne Plus instrument (Life Technologies) using the QuantiFast SyBr Green PCR chemistry (Qiagen), as described previously (Rosati et al., 2011). The reaction mixture for each sample included the SyBr Green mastermix, 8 μL of cDNA and 1.25 mM of each gene-specific primer. The fast PCR amplification included a 5 min activating step at 95°C, and 40 cycles of denaturation (95°C for 10 s) and annealing–extension (60°C for 27 s). Expression values were extracted automatically from the raw fluorescence data using a custom analysis program (Python/PyQt) with a standard threshold crossing algorithm (Ruijter et al., 2013). Multiple different primer pairs were prescreened and only combinations with similar amplification efficiencies were used in the experiments. The 18S and 28S RNAs were used as internal controls, since expression of these did not vary significantly between the samples. Gene expression values were normalized to the average of the 18S and 28S expression values in each sample and expressed as relative values. Connexin 43 and internal control gene expression was analyzed using two gene-specific primer pairs per gene. The expression values obtained with each of the two pairs of primers were very similar and were averaged to obtain the final gene expression values.

The 5′–3′ gene-specific primer sequences were:

Cx43(1) fwd: CGCCGGCTTCACTTTCATTA, rev: TTGTCCAGAAGCTTCCCCAA
Cx43(2) fwd: AGACTGCTTCCTCTCACGTC, rev: AAAGCGAGAGACACCAAGGA
18S(1) fwd: CTCAGCGTGTGCCTACCCTA, rev: GACCCGCACTTACTGGGAT
18S(2) fwd: CGGAACTGAGGCCATGATTA, rev: CTTTCGCTCTGGTCCGTCTT
28S(1) fwd: CTCCGAAGTTTCCCTCAGGA, rev: GGCCCCAAGACCTCTAATCA
28S(2) fwd: AGGACCCGAAAGATGGTGAA, rev: TCGCCCCTATACCCAGGTC

Primers were designed using the Primer3 software (MIT: http://primer3.ut.ee). All primer pairs were validated by electrophoresis analysis of the amplicons and the amplicons were sequenced to verify gene specificity.

## Results

**Figure 1:**
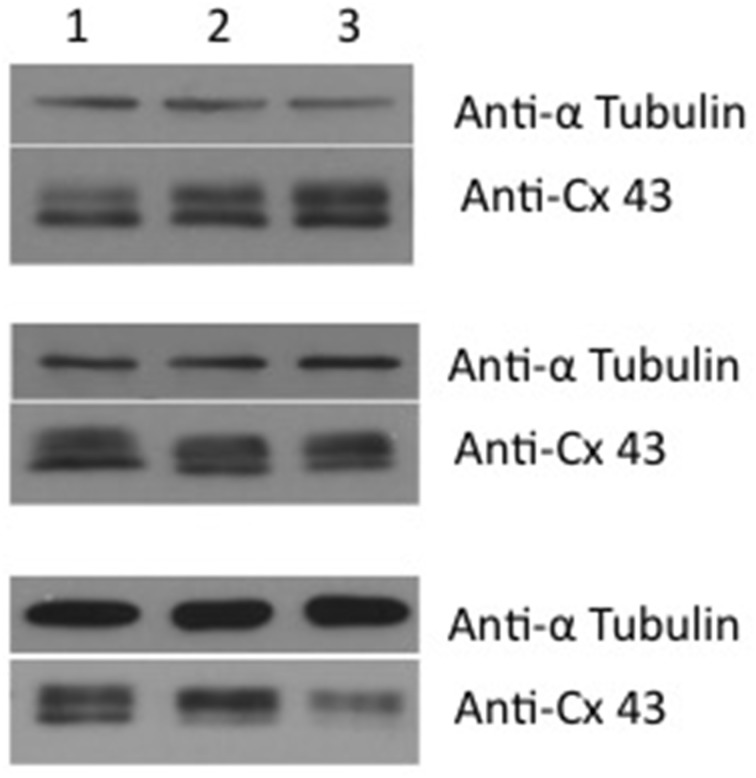
**Western blot of corpora cavernosa smooth muscle cells**. The western blot shows the effect of 4PB after 48 h exposure at concentrations of 0 (lane 1), 1 mM (lane 2), and 5 mM (lane 3). The reporter protein used was tubulin along with Cx43. Three examples are shown. From top to bottom – increased expression, no change and reduced expression. The average value for six Western blots using ImageJ analysis was 0.93 ± 0.25 SD of controls.

**Figure 2:**
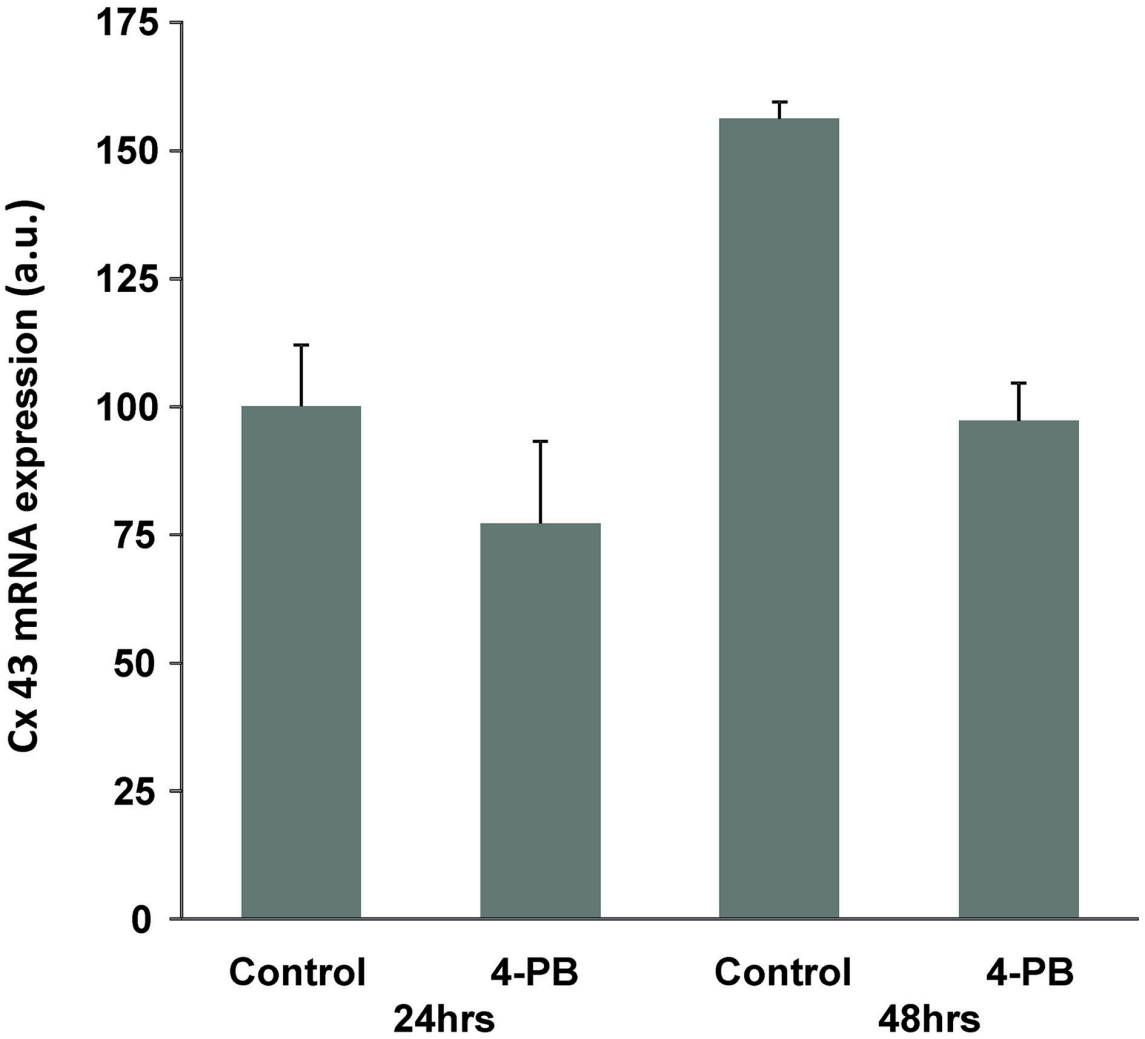
**Effect of 4-PB treatment on Cx43 mRNA expression in corpora cells**. Real-time PCR analysis of the Cx43 gene expression in Frc cells treated with vehicle (Control) and 5 mM of the drug (4-PB) for 24 and 48 h. The columns represent average expression values (normalized to the 24 h control) and the bars are s.e.m. (*n* = 3).

Western blot analysis showed that exposure to 4-PB resulted in no change in Cx43 protein levels in rat corpora smooth muscle cells. Image analysis was performed on six western blots where controls (no 4PB) were compared to 4PB expose preparations. Exposure time to 4PB was 48 h yielding an average value of 0.93 ± 0.25 (SD) for 4PB exposed cells expressing Cx43. Figure 1 compares Cx43 protein levels along with a reporter gene, tubulin from three experiments illustrating the variability of Cx43 expression. In total six Western blot experiments were done and in each case normalized against a control (no 4-PB). 4-PB is not a specific enhancer of expression but rather a general tool able to modify the expression of some but not all proteins within a cell. Neither Cx43 nor tubulin expression was demonstrably affected by 4PB. In other cell systems increases of Cx 43 have been reported (Asklund et al., 2004; Kaufman et al., 2013). Transcription level for Cx43 was also determined. We quantified Cx43 mRNA expression in control and 4-PB treated cells 24 and 48 h after addition of the drug/vehicle (Figure 2). There was a 56% increase in Cx43 mRNA expression in the control corpora cells at 48 h compared to the 24 h controls (*p* < 0.01). Cx43 mRNA expression in the other three cell groups was not significantly different to each other.

The increase in Cx43 mRNA expression with time in culture was prevented by 4-PB treatment, resulting in unchanged Cx43 mRNA levels compared to the 24-h expression level and significantly lower expression relative to the 48-h control cells. Figures 1, 2 taken together suggest that 4-PB, stabilizes Cx43 through an as yet unidentified post-transcriptional mechanism.

**Figure 3:**
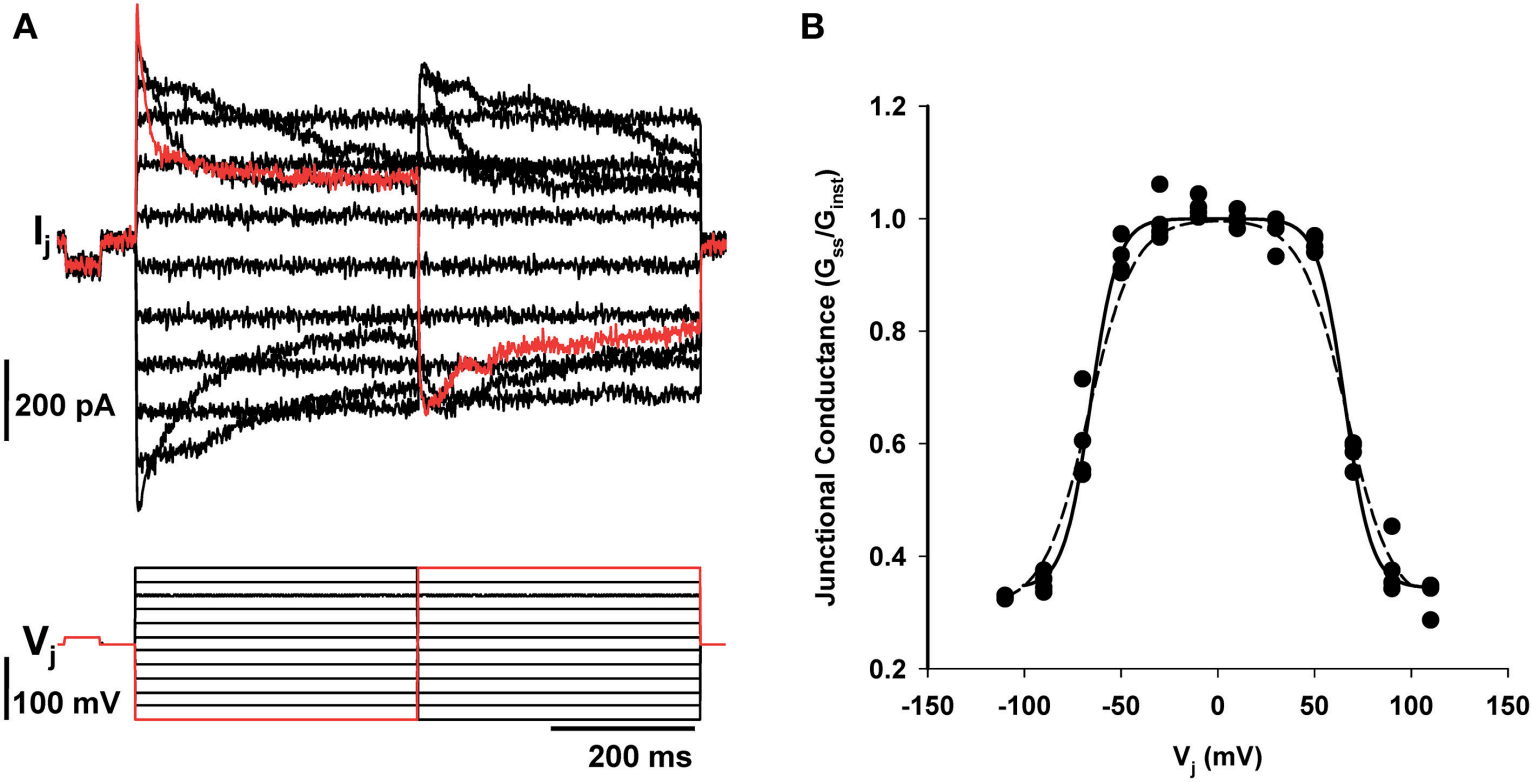
**Dual whole cell patch clamp of corpora cell pair. (A)** Junctional currents recorded in one cell of a pair generated in response to voltage steps in the other. Upper records are junctional current, lower record shows the voltage steps. **(B)** Steady state junctional conductance (G_ss_/G_Ins_*t*) plotted against transjunctional voltage step as previously described (Wang et al., 2010). Filled circles are the data shown in **A**. All data points are from four different cell pairs exposed 4PB (5 mM) for 24 h (*n* = 4). Boltzmans fit of the 4PB data set yielded a *V*_o_ of 67 mV, a *g*_min_ of 0.33. The dashed line represents a Boltzmans fit of control data with the following parameters: *V*_o_ = 67 mV and *g*_min_ = 0.31.

4-PB did not affect the voltage dependence of junctional conductance nor was any change in steady state G_j_ vs. V_j_ observed relative to untreated corpora cell pairs (Rehman et al., 1997; Wang et al., 2001). To demonstrate voltage dependence cell pairs with junctional conductance of 10 nS or less were chosen to minimize error due to series resistance. Figure 3A illustrates an example of junctional current (I_j_) generated in response to transjunctional voltage steps from a cell pair exposed for 48 h to 5 mM of 4-PB. Voltage dependent behavior was identical for 4PB and controls. Figure 3B shows the steady state normalized G_j_–V_j_ relationship for the data shown in Figure 3A and control data as well.

**Figure 4:**
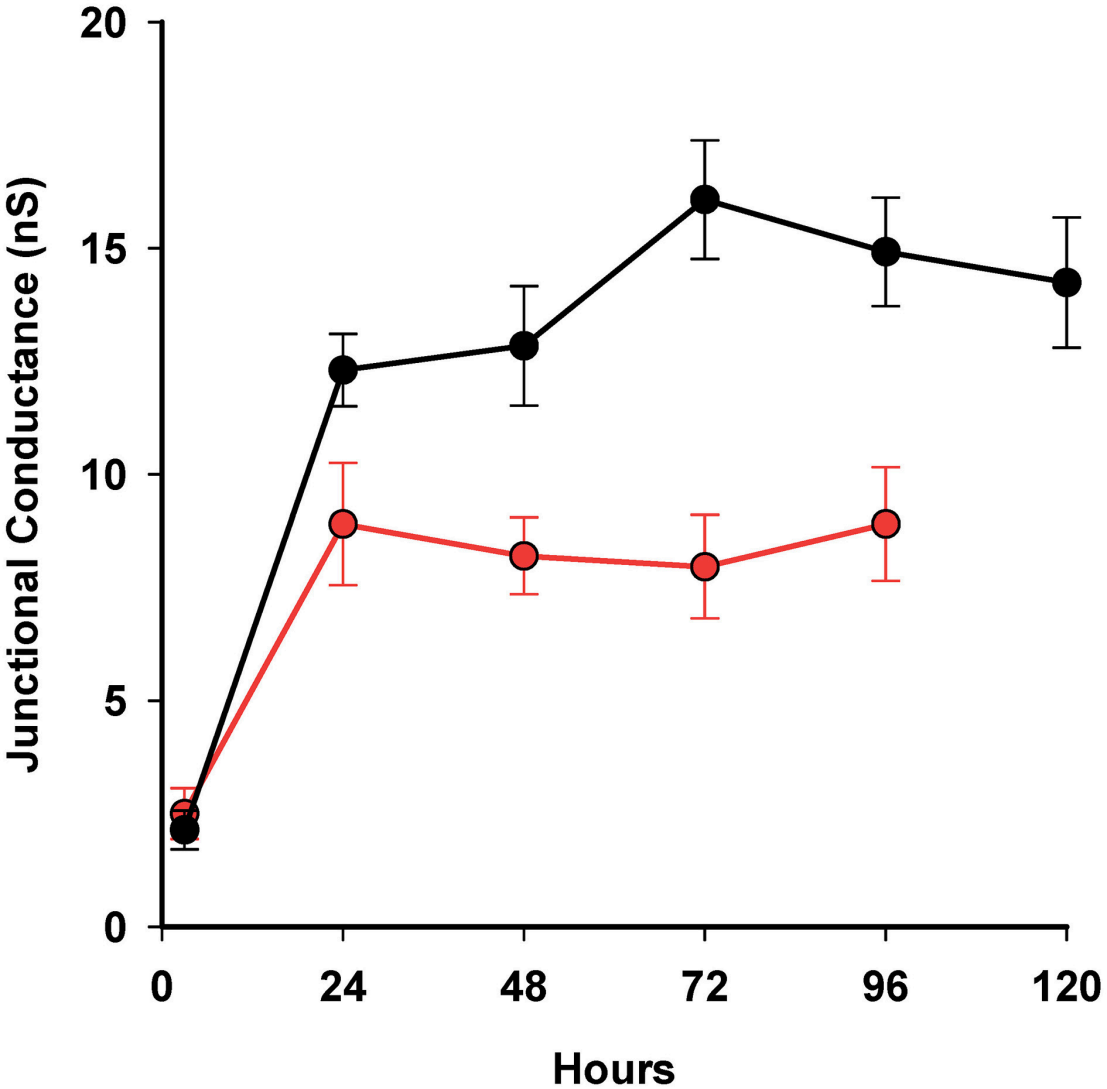
**Summary plot of 4-PB exposed cell pairs and controls over time using a 5 mM concentration**. Three-hour time point: controls *n* = 4, 4PB: *n* = 3; 24 h time point: controls: *n* = 24, 4-PB: *n* = 74; 48 h: controls: *n* = 9, 4PB: *n* = 37; 72 h: controls: *n* = 18, 4-PB: *n* = 38; 96 h controls: *n* = 13, 4PB: *n* = 19. Thirty four cell pairs exposed to 4-PB were studied at 120 h. There was no statistical difference (*T*-test) between controls and 4-PB at 3 h but for 48, 72, and 96 h the *P*-values were all <0.037. Means and standard errors are plotted.

To assess whether 4PB resulted in an increase in junctional conductance it was necessary to generate a database where average junctional conductance was determined at various time points during exposure to 4-PB. The data shown in Figure 4 illustrates that exposure to 4-PB resulted in increased junctional conductance relative to controls. The time course with and without 4-PB over a 5 days period is illustrated. In all cases cells were plated out and then exposed to 5 mM 4-PB or received an equivalent value of media (control) and at various times points dual whole cell patch clamp was performed to assess the magnitude of junctional conductance. The time course to reach an apparent steady state is approximately the same but the extent of coupling is ~2× greater with 4-PB exposure. In transfected HeLa cells and HEK cells 4PB increased junctional conductance ~1.44 over controls (Kaufman et al., 2013). The data are consistent with a stabilization of Cx43 channels such that the degradation rate is slowed relative to the insertion rate. An alternative explanation is an increase in the number of functional channels arising out of a pool of intact but normally silent channels.

## Discussion

These data demonstrate 4-PB exposure results in enhanced junctional conductance (~2X) consistent with previous observations on other cell types (Asklund et al., 2004; Khan et al., 2007; Kaufman et al., 2013). It is also clear from the gene expression data that post-transcriptional mechanisms dominate in determining the final level of functional Cx43 protein expression since mRNA levels are significantly reduced (Figure 2). Up-regulation of Cx43 protein expression and function has been documented in several cell types, by our group and others (Ammerpohl et al., 2004; Asklund et al., 2004; Hattori et al., 2007; Khan et al., 2007; Kaufman et al., 2013). In marked contrast here we find no change in apparent protein expression while Cx43 mRNA expression is decreased following 48-h treatment with 4-PB, as has also been observed by Hattori et al. (2007).

It has been suggested that HDAC inhibitors might increase Cx43 translation or stabilize the existing pool of proteins (Hattori et al., 2007) and our data are consistent with this hypothesis, suggesting that functional Cx43 channels are the end result of broad effects of HDAC inhibition on the network of genes controlling Cx43 translation and post-translational expression.

The apparent down-regulation of Cx43 mRNA at 48 h in culture may also reflect network effects of the drug or could be an example of a weak negative feedback control, by which an increase in Cx43 protein leads to a modest decrease in mRNA transcription.

Another possibility to explain the enhanced junctional conductance is an increased channel open time for a population of channels but this is a less likely explanation based on previous studies using corpora smooth muscle cell pairs where open probability of Cx43 was shown to approach unity (Brink et al., 1996; Christ and Brink, 1999; Wang et al., 2001) even with substantial applied transjunctional potentials. It is also possible that increased junctional conductance is the result of recruitment or activation of intact but silent channels to a functional state (Bukauskas et al., 2000). How 4-PB might trigger such an occurrence is not clear.

The lack of effect on voltage dependence and the steady state G_j_–V_j_ relationship also suggests that expression of other connexins was not significant. The G_j_–V_j_ plot shown in Figure 3 is the same as records previously published for rat corpora cell pairs (Wang et al., 2001) suggesting that the profile of connexin expression was not altered by 4-PB. It is possible that the expression of other connexins is occurring with exposure to 4-PB but the percentage of functional channels other than Cx43 must be small. If there were significant expression of other connexin types the resultant G_j_–V_j_ plot would be different due to the presence of other homotypic channels and/or heterotypic channels (Brink et al., 1997, 2000) We were unable to monitor single channel conductance from cell pairs that were exposed for 24 h or more because the macroscopic junctional conductance was elevated to junctional conductances above 10 nS where it is impossible to directly observe single channel activity. But in a recent study (Kaufman et al., 2013) Cx43 unitary conductance was shown to be unaffected by 4-PB exposure. The data also suggested that there was little or no change in open time probability for Cx43 gap junction channels.

Aging, diabetes, and hypertension all result in reduced expression of Cx43 in animal models and in human males and all three are associated with a higher incidence of erectile dysfunction (Pointis, 2006). Present approaches to alleviate erectile dysfunction center on the therapeutic inhibition of phosphodiesterase V (PDE5) resulting in elevated cGMP concentrations that then trigger relaxation by reducing cytoplasmic calcium levels. 4PB and other related derivatives (Shieh et al., 2012) represent an alternative potential therapy for erectile dysfunction that acts to enhance protein expression by inhibition of HDAC. The use of HDAC inhibitors as a treatment for other conditions such as diabetes and hypertension is also possible if Cx43 expression or other relevant proteins in those diseases can be shown to reduce symptoms (Shieh et al., 2012).

### Conflict of interest statement

The authors declare that the research was conducted in the absence of any commercial or financial relationships that could be construed as a potential conflict of interest.
